# Chronic angioedema caused by navel orange but not citrus allergy: case report

**DOI:** 10.1186/2045-7022-3-S3-P159

**Published:** 2013-07-25

**Authors:** S Boonpiyathad

**Affiliations:** 1 Department of medicine, Phramonkutklao Hospital, Bangkok, Thailand

## Background

Orange allergy is uncommon in general population. Most patients had Immunoglobulin E (IgE) mediated symptoms. There are three types of orange allergens which can be classified as germin-like protein (Cit s1), profiling (Cit s2) and lipid transfer protein (Cit s3).

## Methods

We reported a case of angioedema diagnosed by oral challenge test, but not by skin prick test and serum specific IgE.

## Results

### Case

A 30-year-old Thai woman, who presented with a history of angioedema for 6 months. Her angioedema involved lip, eyelid and hand swelling. She was referred to our Allergy Clinic for further investigation. It was found that her symptoms of angioedema usually occurred in 30 minutes after ingesting navel orange and disappeared after 1-2 hours later. She favored in navel orange and she had eaten 1 kilogram of navel oranges per day for 8 years. The results of laboratory tests, including complete blood count, thyroid function test, complement level and autologous serum skin test showed normal values. The results of skin prick testing were negative to aero and food allergen, including orange. The level of serum IgE was 16 kU/L, while the level of specific IgE for Cit s1, Cit s2 and Cit s3 were 0. Prick to prick test with 6 orange types; navel orange, sunkist orange, murcott orange, mandarin orange, tangerine and pomelo showed negative results. Oral food challenge test with 6 orange types, lemon and grapefruit revealed that angioedema occurred after eating one segment of navel orange. Therefore, she was advised to avoid navel orange and her angioedema was vanished.

## Conclusion

Culprit navel orange allergen is undetected. Sometime skin prick tests and specific IgE could not be used to help in food allergy diagnosis. In contrast, Oral food challenge test still has remained the goal standard for food allergy diagnosis.

## Disclosure of interest

None declared.

